# Predictors of Submaximal Exercise Test Attainment in Adults Reporting Long COVID Symptoms

**DOI:** 10.3390/jcm11092376

**Published:** 2022-04-23

**Authors:** Roman Romero-Ortuno, Glenn Jennings, Feng Xue, Eoin Duggan, John Gormley, Ann Monaghan

**Affiliations:** School of Medicine, Trinity College Dublin, D02 R590 Dublin, Ireland; gljennin@tcd.ie (G.J.); fexue@tcd.ie (F.X.); dugganeo@tcd.ie (E.D.); jgormley@tcd.ie (J.G.); ann.monaghan@tcd.ie (A.M.)

**Keywords:** long COVID, exercise tolerance, cardiopulmonary exercise test, heart rate, observational study

## Abstract

Adults with long COVID often report intolerance to exercise. Cardiopulmonary exercise testing (CPET) has been used in many settings to measure exercise ability but has been conducted in a few long COVID cohorts. We conducted CPET in a sample of adults reporting long COVID symptoms using a submaximal cycle ergometer protocol. We studied pre-exercise predictors of achieving 85% of the age-predicted maximum heart rate (85%HRmax) using logistic regression. Eighty participants were included (mean age 46 years, range 25–78, 71% women). Forty participants (50%) did not reach 85%HRmax. On average, non-achievers reached 84% of their predicted 85%HRmax. No adverse events occurred. Participants who did not achieve 85%HRmax were older (*p* < 0.001), had more recent COVID-19 illness (*p* = 0.012) with higher frequency of hospitalization (*p* = 0.025), and had been more affected by dizziness (*p* = 0.041) and joint pain (*p* = 0.028). In the logistic regression model including age, body mass index, time since COVID-19, COVID-19-related hospitalization, dizziness, joint pain, pre-existing cardiopulmonary disease, and use of beta blockers, independent predictors of achieving 85%HRmax were younger age (*p* = 0.001) and longer time since COVID-19 (*p* = 0.008). Our cross-sectional findings suggest that exercise tolerance in adults with long COVID has potential to improve over time. Longitudinal research should assess the extent to which this may occur and its mechanisms. ClinicalTrials.gov identifier: NCT05027724 (TROPIC Study).

## 1. Introduction

On 11 March 2020, the World Health Organization (WHO, Geneva, Switzerland) declared the SARS-CoV-2 outbreak a global pandemic [1]. At the time of writing, COVID-19 has claimed in excess of 5.7 million deaths worldwide, but the cumulative number of cases is almost 68 times higher [2]. Among COVID-19 survivors, many have developed the ‘post COVID-19 condition’, “which occurs in individuals with a history of probable or confirmed SARS-CoV-2 infection, usually 3 months from the onset of COVID-19 with symptoms that last for at least 2 months and cannot be explained by an alternative diagnosis” [3]. This post COVID-19 condition, also known as ‘long COVID’, has an unknown global prevalence but up to 1 in 3 individuals may have one or more symptoms at 3 to 6 months after COVID-19 diagnosis [4]. Among a constellation of possible symptoms, adults with long COVID often report ongoing fatigue and intolerance to exercise [5,6,7]. Research has shown that higher initial illness acuity (e.g., emergency room visit) is a risk factor for both chronic fatigue and reduced exercise tolerance after COVID-19 [8]. It has been proposed that long COVID may be a state of reduced fitness mainly caused by muscle deconditioning [9,10,11]. However, others have argued that the mechanisms of exercise intolerance after COVID-19 may be more complex and involve metabolic and cardiopulmonary pathways [12]. The extent to which post-COVID-19 exercise intolerance may improve over time is poorly understood.

Cardiopulmonary exercise testing (CPET) has been used in many settings to evaluate exercise intolerance and objectively determine functional capacity and impairment [13]. CPET has been used in a range of cardiovascular and non-cardiovascular conditions for diagnosis and risk stratification [14]. The criterion assessment for cardiorespiratory fitness requires subjects to undergo a physical challenge of continuously increasing workload to maximal exertion, with the goal of reaching a state of plateaued oxygen consumption, despite this increased workload, to determine the subjects’ maximal capacity for oxygen consumption (VO_2max_). However, in potentially vulnerable clinical populations for whom such a test could prompt an adverse response, CPET is often terminated at a pre-determined submaximal level (e.g., when subjects reach 85% of their age-predicted maximal heart rate (85%HRmax)), which rules out significant chronotropic incompetence [15] and confers a better prognosis [16]. Despite its potential utility to investigate exercise intolerance states, CPET has to date been conducted in only a few long COVID cohorts [17]. This study examined correlates and baseline (pre-exercise) predictors of achieving 85%HRmax in a sample of adults reporting long COVID symptoms.

## 2. Materials and Methods

### 2.1. Study and Cohort Description

This was a cross-sectional observational study on a participant cohort recruited for the TROPIC (Technology assisted solutions for the Recognition of Objective Physiological Indicators of post-Coronavirus-19 fatigue) investigation at Trinity College Dublin and St James’s Hospital Dublin, Ireland. The study received full ethical and regulatory approvals. All participants provided written informed consent to partake in the study. Participants were eligible for inclusion under all the following criteria: (1) age 18 years or older; (2) self-reported history of SARS-CoV-2 infection; (3) experiencing prolonged symptoms such as fatigue; (4) free from absolute contraindications to exercise [18,19]; and (5) able to provide informed consent. Participants were recruited from the following sources in our hospital: (1) falls and syncope unit; (2) geriatric day hospital; (3) post COVID-19 outpatient clinic; (4) staff who had contracted COVID-19; and (5) participants from earlier post-COVID-19 research who had consented to be contacted for further studies. In addition, we also considered (6) self-referrals. COVID-19 and non-COVID-19 exclusion criteria for enrolment have been reported elsewhere [20].

### 2.2. Participant Characteristics

The following participant characteristics were collected:

○ Demographics: age, sex.
○ Anthropometrics: body mass index (BMI, Kg/m^2^).
○ Days since COVID-19 illness, and whether a participant was hospitalized or not due to COVID-19 (at least one overnight stay).
○ Cardiopulmonary disease prior to COVID-19 (chronic respiratory disease, chronic heart disease, or hypertension: yes or no).
○ Current medications: antihypertensives, beta blockers (yes or no).
○ Smoking status: never smoked versus former/current smoker.
○ Resting seated systolic (SBP), diastolic (DBP) blood pressure and HR were measured prior to CPET with an oscillometric brachial blood pressure measurement device (Connex^®^ Vital Signs Monitor, Welch Allyn Inc., Skaneateles Falls, NY, USA).
○ Long COVID symptomatology. Participants were asked if following COVID-19 illness they had ongoing symptoms (yes or no) of: shortness of breath, cough, throat pain, chest tightness, chest pain, heart palpitations, headache, loss of smell, loss of taste, brain fog, sleeping problems, dizziness, muscular weakness, muscular pain, or joint pain.
○ Participants were also administered the 11-item Chalder Fatigue Scale (CFQ), a self-rating scale developed to measure the severity of physical and mental fatigue [21]. We employed the Likert scoring system, with an overall scale range from 0 (minimum) to 33 (maximum fatigue).

### 2.3. CPET Protocol

CPETs were conducted using a cycle ergometer (COSMED E100, COSMED SRL, Rome, Italy) in an exercise physiology laboratory kept at 20 °C under medical supervision. Best practice recommendations for CPET testing during the COVID-19 pandemic were followed [22]. Predicted maximum HR was calculated as 208 − 0.7 × age [23]. We also calculated predicted VO_2max_, by plotting breath-by-breath VO_2_ values (excluding 3-min warm up) on y axis and HR on the x axis, applying a line of best fit to VO_2_ values and extrapolating a comparator y value for x = predicted maximum HR. VO_2_ values were cleaned to exclude aberrations arising from coughs, sighs, etc., by the COSMED proprietary software (COSMED CPET Suite, V10.0E). We adjusted the height of the seat individually prior to each assessment, to a position at which each of the participants agreed that he/she was comfortable to carry out the assessment. CPET testing commenced with 3 min of free wheel pedaling after which resistance increased by standardized 10, 15, 20, or 25 Watt 1-min increments (depending on age, sex, gender, and weight). Breath-by-breath gas analysis (COSMED K4b2) and heart rate (POLAR H10 chest band) were continuously measured. Peripheral oxygen saturation via pulse oximetry (SpO_2_%), rate of perceived exertion (modified Borg scale: 0 minimum–10 maximum) [24], and self-selected pedaling frequency (cadence) in revolutions per minute (rpm) were measured at regular 1-min intervals. The primary test termination criterion was 85%HRmax. The test was terminated early if the pedal frequency dropped below 40 rpm, the participant requested termination due to excessive fatigue, or any adverse event occurred.

For descriptive purposes, the following CPET performance parameters were noted:

○ Test duration (seconds).
○ Peak HR achieved (beats per minute, bpm).
○ Peak work rate (WR, Watts).
○ Peak oxygen consumption (VO_2_peak), average during last 30 s of exercise, per Kg (mL/Kg/min).
○ VE/VCO_2_ (ventilatory efficiency) [25,26], average during last 30 s of exercise.
○ Lowest SpO2% value.
○ Maximum Borg score.

### 2.4. Statistical Analyses

Statistics were computed with IBM^®^ SPSS^®^ Statistics for Windows, Version 26.0, Armonk, NY, USA: IBM Corp. Descriptives were given with count and percentage (%), mean with standard deviation (SD), median with interquartile range (IQR), and range. We utilized the SPSS Chart Builder to visualize CPET features between 85%HRmax groups via cluster line chart with representation of 95% confidence intervals (CI) around means and interpolation of missing data. In each of the two 85%HRmax groups, Shapiro-Wilk tests with visual assessment of histograms and Q-Q plots were used to assess normality of continuous variables. Following this, to compare characteristics between groups, we utilized the non-parametric 2-sided Mann-Whitney U test for continuous variables, and the Chi-square test for dichotomous characteristics. To study independent predictors of dichotomous group membership (85%HRmax achieved versus not achieved), we computed a backwards logistic regression model, and for each predictor extracted the Odds Ratio (OR) and 95% CI for the OR. Multicollinearity checks were conducted. Statistical significance was defined as *p* < 0.05 throughout.

In an attempt to reduce between-group heterogeneity for age and days since COVID-19, a second backwards logistic regression model was computed as a sensitivity analysis including only participants who were >12 weeks (90 or more days) post-COVID-19 [27] and aged 35 years or older.

### 2.5. Ethical Approval

This study received full approval by the St James’s Hospital/Tallaght University Hospital Joint Research Ethics Committee (Submission Number: 104: TROPIC; Approval Date: 4 May 2021) and the St James’s Hospital Research & Innovation Office (Reference: 6566; Approval Date: 14 May 2021). The study was performed in accordance with the 1964 Declaration of Helsinki and its later amendments. All participants gave their informed consent prior to their inclusion in the study.

## 3. Results

Eighty participants were included (mean age 46 years, range 25–78, 71% women). Eleven participants were aged less than 35 years, three of whom did not attain 85%HRmax. Mean BMI was 27.7 Kg/m^2^. The median time post-acute COVID-19 illness was 320 days (range 39–655); only two participants were less than 90 days since COVID-19, neither of whom attained 85%HRmax. Overall, 17.6% of the participants had been hospitalized. Eleven participants (13.8%) had pre-existing respiratory disease (asthma in 10, obstructive sleep apnoea in 1); chronic heart disease was present in 2 participants (both atrial fibrillation without symptomatic heart failure or ischaemic heart disease), and 14 (17.5%) had hypertension and were taking at least one antihypertensive. Eight participants (10%) were taking beta blockers. Forty participants (50%) never smoked. Mean (SD) seated SBP, DBP, and HR prior to CPET was 130.2 (16.1) mmHg, 80.7 (11.0) mmHg, and 71.2 (10.8) bpm, respectively. The median CFQ score for the total sample was 25 (range 13–33). Forty participants (50%) reached 85%HRmax. The other 40 participants requested termination due to fatigue. No adverse events occurred. Table 1 shows the comparison of participants’ characteristics by 85%HRmax attainment, including long COVID symptomatology. Participants who did not achieve 85%HRmax were older (*p* < 0.001) and had more recent COVID-19 illness (*p* = 0.012) with higher frequency of hospitalization (*p* = 0.025) and had been more affected by dizziness (*p* = 0.041) and joint pain (*p* = 0.028).

**Figure 1:**
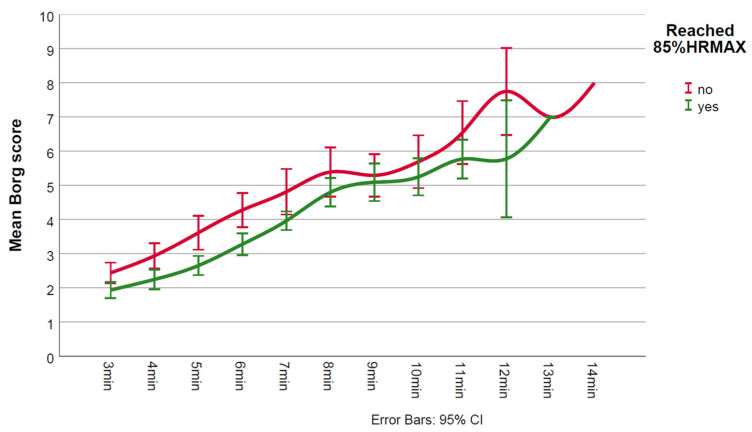
Mean in-exercise Borg values for achievers and non-achievers.

**Figure 2:**
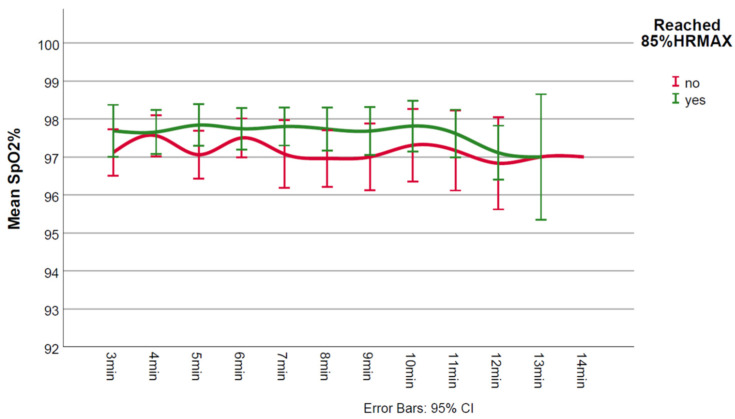
Mean in-exercise SpO_2_% values for achievers and non-achievers.

**Figure 3:**
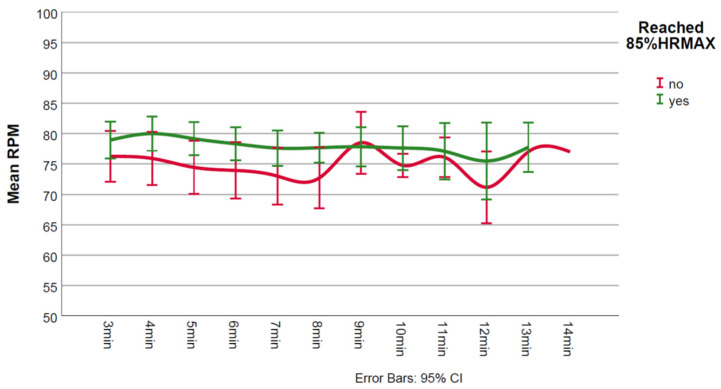
Mean revolutions per minute (rpm) values for achievers and non-achievers.

During CPET, non-achievers had lower test duration (*p* = 0.008), lower work rate (*p* = 0.002), lower VO_2_peak (*p* < 0.001), lower ventilatory efficiency (*p* = 0.007), and higher maximum Borg score (*p* = 0.026) (Table 1). Figure 1, Figure 2 and Figure 3 show the in-exercise mean values for Borg, SpO_2_% and rpm, respectively.

In the backwards logistic regression model including age, body mass index, time since COVID-19, COVID-19-related hospitalization, dizziness, joint pain, pre-existing cardiopulmonary disease, and use of beta blockers, independent predictors of achieving 85%HRmax were younger age (*p* = 0.001) and longer time since COVID-19 (*p* = 0.008) (Table 2). The second logistic regression model including the 68 participants who were aged 35 and over and at least 90 days post-COVID-19 is shown in Appendix A. Results for age and time since COVID-19 remained unchanged, and an additional significant effect of hospitalization was shown.

## 4. Discussion

In this observational cross-sectional study, we conducted CPET in a sample of adults reporting long COVID symptoms and investigated correlates and baseline (pre-exercise) predictors of achieving 85%HRmax. We found that younger age and longer time since COVID-19 were independently predictive of 85%HRmax attainment. Overall, the sample reported significant fatigue at baseline, with median CFQ score of 25 out of maximum of 33. Our study underscores both the feasibility and safety of our submaximal CPET protocol in this highly symptomatic sample including a wide age range (25–78), 18% prevalence of treated hypertension, 14% of pre-existing respiratory disease, and where 18% had been previously hospitalized for COVID-19.

While mean predicted 85%HRmax was not too dissimilar for non-achievers and achievers (147 vs. 152 bpm, respectively, Table 1), on average non-achievers reached 84% (124 bpm) of their predicted 85%HRmax. Non-achievers seemed to experience higher intensity of perceived exertion during the test as evidenced by higher maximum Borg scores. Judging by the lack of overlap between 95% CIs, it appeared that lower mean Borg scores were present in achievers during minutes 5 and 6 (Figure 1). Specifically, baseline SpO_2_% values were normal and there were no significant desaturations (≥3%) during exercise in any of the two groups (Figure 2). Mean minute-by-minute rpm did not seem different between groups (Figure 3).

Older age has been reported as a risk factor for both COVID-19 related hospitalization [28] and hospital-associated functional loss during a COVID-19-related admission [29,30]. In a study of 100 adults (mean age 47 years) who were 3–6 months after COVID-19 diagnosis, it was found that exercise capacity as measured by VO_2_peak was reduced in those with severe COVID-19 disease, defined as hospitalization with or without intensive care unit/non-invasive ventilation [31]. In another study with 200 participants (mean age 49), those with post-COVID-19 syndrome showed significantly lower VO_2_peak as compared to asymptomatic subjects [32]. In 70 post-COVID-19 patients (mean age 55), Aparisi et al., showed that those reporting persistent dyspnea had a significantly reduced predicted VO_2_peak consumption [33]. In 10 patients (mean age 50), Mohr et al., showed that while mean peak work rate was preserved, mean peak oxygen uptake was reduced, but on chest computed tomography, 6 patients had abnormalities and mean diffusion capacity of the lung for carbon monoxide was slightly reduced in the cohort [34]. A study with 205 participants (mean age 38) where 25% were classified as having dysautonomia (resting HR > 75 bpm, HR increase with exercise < 89 bpm, and HR recovery < 25 bpm 1 min after exercise), participants with dysautonomia demonstrated a significantly reduced peak oxygen consumption on CPET [35]. With only two participants reaching <89 bpm peak HR (one on beta blocker), dysautonomia was not significantly prevalent in our cohort [20].

COVID-19 higher acuity with need for hospitalization (often displaying thromboinflammatory features [36], cytokine storm [37], and/or requiring non-invasive and/or invasive ventilation) may be associated with greater cardiopulmonary abnormalities and hence more prolonged post-COVID-19 symptomatology [38,39,40]. As one study suggested, objective respiratory, functional, radiological, and cognitive abnormalities were more prominent in hospitalized patients [41]. Another study showed that at 6 months, those hospitalized in the general ward had a relatively preserved VO_2_peak, whereas those who had been in the intensive care unit had a moderately reduced VO_2_peak [42]. In 24 post-COVID-19 patients who had been mechanically ventilated in intensive care, cardiorespiratory fitness was very poor with a median peak oxygen uptake of 57% of predicted values [43]. In a study of previously hospitalized COVID-19 patients, Cassar et al., found that at 2–3 months, 55% had reduced VO_2_peak on CPET secondary to symptomatic limitation and muscular impairment; however, by 6 months, the proportion had reduced to 31% [44]. This seems consistent with our finding that the longer time post-COVID-19, the more likely it was for participants to achieve the 85%HRmax CPET target. In our series, only one of the 80 participants (an 85%HRmax non-achiever) was admitted to the intensive care unit (ICU) during COVID-19 hospitalization, so it was not possible to statistically study if ICU or mechanical ventilation during COVID-19 hospitalization, and not only the hospitalization itself, influenced exercise performance during the long COVID phase.

There have also been negative CPET studies. In 18 participants (mean age 41), one study showed that compared to matched controls, participants with post-acute sequelae of SARS-CoV-2 infection (PASC) had similar peak oxygen consumption [45]. Another negative study in 75 participants (mean age 57) showed that initial disease severity did not impact on exercise capacity in COVID-19 survivors at 3 months after hospital discharge, including a ventilatory response in the normal limit [46]. Some CPET studies with athletes have also been negative. For example, in athlete swimmers who had had mild COVID infection, CPET performance was no different from controls [47]. Similar negative findings were reported in another study with 90 competitive athletes, most of whom were mildly symptomatic [48]. Komici et al., showed that in athletes with post-COVID-19 symptoms (e.g., anosmia, ageusia), VO_2_peak was not significantly different than controls [49]. In another study with 13 elite cross-country skiers with previous mild-moderate COVID-19 symptoms, it was shown that cases reached the aerobic threshold earlier than controls, whereas the remaining CPET parameters did not differ between groups [50].

From the literature, we can theorize that post-COVID-19 cohorts may have heterogeneous drivers of lower CPET performance; for example, in some individuals, poor performance may be primarily driven by residual cardiopulmonary dysfunction; whilst in others, it may be due to neuromuscular impairments. However, it is possible, and entirely plausible, that in some individuals there are mixed cardiopulmonary, neuromuscular, and/or functional drivers. For example, in a study with 200 participants (median age 59), 50% had median percent-predicted peak oxygen uptake (%pVO_2_) below the 85% predicted value, but 51% of those had non-cardiopulmonary reasons for exercise limitation [51]. In a study by Mancini et al., that included 41 long COVID patients at a mean of 9 months after COVID-19, it was suggested that dysfunctional breathing and resting hypocapnia could contribute to symptoms [52]. Furthermore, in a study using invasive cardiopulmonary exercise testing (iCPET), Singh et al., showed that compared to 10 controls, 10 cases (mean age 48) who had recovered from COVID-19 without cardiopulmonary disease but persistent exertional and functional limitation approximately 11 months after acute viral illness, exhibited markedly reduced VO_2_peak from a peripheral rather than a central cardiac limit, along with an exaggerated hyperventilatory response during exercise [53]. Indeed, hyperventilation has been cited as a possible explanation for long-lasting exercise intolerance in mild COVID-19 survivors [54]. In addition, the potential importance of contextual/test-related factors affecting CPET performance was suggested by a study where cloth face masks led to a 14% reduction in exercise time and 29% decrease in VO_2_max, which the authors attributed to perceived discomfort associated with mask-wearing [55].

Our study has important limitations. Firstly, regarding the reason for early test termination, we only recorded it as global perceived fatigue, rather than probing participants further to identify if it was predominantly musculoskeletal or ventilatory in nature. In contrast with several other CPET studies, we did not have access to cardiac or lung imaging, lung function tests or blood tests such as lactate, NT-proBNP or creatinine kinase (CK). We conducted a cross-sectional observational study with an internal control design (achievers vs. non-achievers), but we did not have an external control sample. Generalizability cannot be assumed from our design. In addition, with only 40 participants in each group, our statistical power was limited, particularly given the wide heterogeneity of the study participants in terms of age (achievers being a mean of 8 years younger) and days post-COVID-19 (achievers being a median of 7 months later), despite the robustness of the sensitivity analysis. Also because of this, we did not a priori exclude participants with pre-existing respiratory or cardiac disease, or those on HR-modifying medications; however, these numbers were low and not significantly different between groups (Table 1). In addition, also due to limited power, we could not include more predictors in the regression model. For improved characterization of participants, a survey of fitness status before COVID-19 infection would have been interesting (e.g., hours of sport/week); however, we did not collect this information.

In conclusion, 50% of our long COVID participants did not achieve 85%HRmax. Long COVID is likely to be a heterogeneous condition influenced by both medical and functional factors, and both may affect CPET performance. Whilst impaired pulmonary, cardiac, and/or musculoskeletal muscle function may primarily contribute to the limitation of performance in some post-COVID-19 patients (especially in those chronologically closer to time of hospitalization), in other patients, neuromuscular factors and/or functional limitations may be more important drivers. Our research cannot shed light on the exact mechanisms responsible for the limited exercise tolerance in half of our sample; however, our observations are in keeping with the possibility that reduced fitness in long COVID may improve over time. To disentangle this complexity, longitudinal research with comprehensively characterized cohorts is warranted to see the extent to which drivers and fitness levels evolve over time in adults with long COVID. Indeed, CPET can be part of a diagnostic pathway and longitudinal follow up for long COVID patients [56].

## Figures and Tables

**Table 1:** Participant characteristics by achievement of 85% maximum predicted heart rate.

Characteristic	Did Not Reach 85% Maximum HR (*n* = 40)	Reached 85% Maximum HR (*n* = 40)	*p* for Difference
Baseline characteristics			
Mean age, years (SD)	50.0 (9.7)	42.0 (7.6)	<0.001 ^^^
Female sex (%)	72.5	70.0	0.805 ^+^
Mean BMI, Kg/m^2^ (SD)	29.0 (5.4)	26.3 (4.2)	0.050 ^^^
Median days post-COVID-19 (IQR)	256.5 (294.5)	464.0 (288.0)	0.012 ^^^
Hospitalized during COVID-19 illness (%)	27.8	7.9	0.025 ^+^
Admitted to ICU (%)	2.5	0.0	1.000 ^++^
Previous respiratory disease (%)	17.5	10.0	0.330 ^+^
Previous heart disease (%)	5.0	0.0	0.247 ^++^
Treated hypertension (%)	22.5	12.5	0.239 ^+^
Cardiopulmonary disease (%)	40.0	20.0	0.051 ^+^
On beta blockers (%)	15.0	7.5	0.481 ^++^
Never smoked (%)	47.2	62.2	0.200 ^+^
Mean seated SBP, mmHg (SD)	132.3 (18.2)	128.0 (13.5)	0.503 ^^^
Mean seated DBP, mmHg (SD)	80.3 (13.5)	81.1 (7.9)	0.972 ^^^
Mean resting HR, bpm (SD)	69.6 (10.3)	72.8 (11.1)	0.320 ^^^
Long COVID symptomatology			
Shortness of breath (%)	75.7	71.1	0.651 ^+^
Cough (%)	21.6	26.3	0.634 ^+^
Throat pain (%)	37.8	28.9	0.414 ^+^
Chest tightness (%)	56.8	55.3	0.896 ^+^
Chest pain (%)	32.4	36.8	0.688 ^+^
Heart palpitations (%)	59.5	55.3	0.713 ^+^
Headache (%)	62.2	73.7	0.285 ^+^
Loss of smell (%)	21.6	13.2	0.333 ^+^
Loss of taste (%)	18.9	13.2	0.496 ^+^
Brain fog (%)	56.8	71.1	0.197 ^+^
Sleeping problems (%)	73.0	55.3	0.110 ^+^
Dizziness (%)	73.0	50.0	0.041 ^+^
Muscular weakness (%)	10.8	7.9	0.711 ^++^
Muscular pain (%)	59.5	47.4	0.294 ^+^
Joint pain (%)	59.5	34.2	0.028 ^+^
Median CFQ score (IQR)	25.5 (9.3)	24.5 (8.0)	0.711 ^^^
CPET characteristics			
Mean predicted 85%HRmax, bpm (SD)	147.1 (5.8)	151.8 (4.5)	<0.001 ^^^
Mean predicted VO_2_ max, mL/Kg/min (SD)	29.4 (7.1)	29.8 (8.4)	0.761 ^^^
Mean CPET duration, seconds (SD)	402.1 (136.4)	469.2 (96.2)	0.008 ^^^
Mean peak HR achieved during CPET, bpm (SD)	123.5 (16.7)	153.7 (5.4)	<0.001 ^^^
Mean peak work rate achieved during CPET, W (SD)	96.0 (40.1)	133.5 (57.6)	0.002 ^^^
Mean peak VO_2_ achieved during last 30 s of exercise per Kg, mL/Kg/min (SD)	16.1 (5.5)	21.8 (7.2)	<0.001 ^^^
Mean VE/VCO_2_ during last 30 s of exercise (SD)	33.2 (5.5)	29.8 (4.6)	0.007 ^^^
Mean lowest SpO_2_% during CPET (SD)	96.0 (1.9)	96.7 (1.8)	0.128 ^^^
Mean maximum Borg score during CPET (SD)	6.7 (1.6)	5.9 (1.4)	0.026 ^^^

^^^ Independent samples Mann-Whitney U test; ^+^ Chi-square test; ^++^ 2-sided Fisher’s exact test. HR: heart rate; n: number; SD: standard deviation; BMI: body mass index; ICU: intensive care unit; IQR: interquartile range; CFQ: Chalder fatigue questionnaire score; SBP: systolic blood pressure; DBP: diastolic blood pressure; bpm: beats per minute; CPET: cardiopulmonary exercise test; W: Watts.

**Table 2:** Logistic regression to predict achievement of 85% maximum predicted HR during exercise.

	Odds Ratio	95% Confidence Interval for Odds Ratio		*p*
		Lower	Upper	
Age	0.879	0.815	0.949	0.001
Days post-COVID-19	1.006	1.001	1.010	0.008
Hospitalized	0.227	0.040	1.297	0.095
Joint pain	0.372	0.118	1.177	0.093

The model converged in 5 steps. Variables entered on step 1: age, body mass index, cardiopulmonary disease, beta blocker, days since COVID-19, hospitalized, dizziness, joint pain.

## Data Availability

The datasets generated and analyzed for this study are not publicly available due to ethical approval reasons.

## Appendix A

**Table A1:** Sensitivity analysis. Logistic regression to predict achievement of 85% maximum predicted HR during exercise (N = 68 participants who were aged 35 and over and at least 90 days post-COVID-19). The model converged in 6 steps. Variables entered on step 1: age, body mass index, cardiopulmonary disease, beta blocker, days since COVID-19, hospitalized, dizziness, joint pain.

	Odds Ratio	95% Confidence Interval for Odds Ratio		*p*
		Lower	Upper	
Age	0.861	0.776	0.955	0.005
Days post-COVID-19	1.006	1.001	1.010	0.014
Hospitalized	0.119	0.017	0.816	0.030
